# Chronic Schizophrenia Presenting With Psychogenic Polydipsia Concealing Stage IV Uterine Adenocarcinoma: A Case Report

**DOI:** 10.7759/cureus.66752

**Published:** 2024-08-13

**Authors:** Kamayel Jaludi, Dakota Pastore, Angelo Sica

**Affiliations:** 1 Osteopathic Medicine, Rowan-Virtua School of Osteopathic Medicine, Stratford, USA; 2 General Surgery, Rowan-Virtua School of Osteopathic Medicine, Stratford, USA; 3 Psychiatry and Behavioral Sciences, St. Joseph’s Medical Center, Paterson, USA

**Keywords:** bioethical issue, mental health, healthcare disparities, uterine neoplasms, adenocarcinoma, disparities, uterine adenocarcinoma, hyponatremia, schizophrenia, psychogenic polydipsia

## Abstract

Psychogenic polydipsia (PPD) may be commonly seen in patients suffering from schizophrenia. It remains unknown how often psychiatric illness can mask other more serious conditions. The patient is a 58-year-old female with chronic schizophrenia and PPD presenting to the emergency department (ED) with abdominal pain over a seven-year period from 2016 to 2022 with her symptoms attributed to a schizophrenia exacerbation with minimal to no diagnostic follow-up. After several ED admissions, in 2022, tumor marker tests were collected yielding concerning results for underlying cancer including CA125 85.9/50.1, CA19-9 >10, and CEA 0.3. A pelvic ultrasound was completed in 2022 after another three ED visits, revealing an infiltrative uterine mass measuring up to 5.6 cm, which was confirmed by CT abdomen and pelvis to be stage IV uterine adenocarcinoma. Several potential opportunities for intervention were missed in this patient including (1) primary prevention, (2) inadequate physical exam and history acquisition, and (3) delayed diagnostic imaging from the onset of abdominal pain to diagnosis. This case highlights the shortcomings across disciplines in providing early intervention and the disparities of basic patient care in psychiatric patients.

## Introduction

Schizophrenia is a chronic and severe mental disorder characterized by disturbances in thought processes, perceptions, emotions, and behavior, often causing significant distress for the patient and their family [1]. Although the prevalence of schizophrenia in the general population is estimated at 1%, the incidence of schizophrenia diagnosis has been increasing [1]. The exact cause of schizophrenia is not fully understood but is believed to involve a combination of genetic, biological, and environmental factors [2]. It has been established that various neurotransmitters have been involved in the development of schizophrenia and that certain drugs may accelerate a schizophrenia diagnosis in a patient with a high predisposition [2,3]. This burdensome disorder is often diagnosed in early adulthood and results in impaired social and occupational functioning [1-2]. Moreover, schizophrenia is considered a diagnosis of exclusion and is based on the Diagnostic and Statistical Manual of Mental Disorders, Fifth Edition (DSM-5) criteria, which require symptoms to be present for a duration of at least six months [1-2]. These criteria include positive symptoms such as delusions, hallucinations, disorganized speech and behavior, and negative symptoms described as social isolation, decreased emotional expression, and processing speed. Unfortunately, schizophrenia is associated with reduced life expectancy with a lifetime suicide risk ranging between 5% and 10% [4]. 

Schizophrenia can be complicated with psychogenic polydipsia (PPD), also known as primary polydipsia, a disorder characterized by excessive water intake and water-seeking behavior due to psychological rather than physiological needs [5]. Poorly controlled schizophrenia could potentially lead to thoughts encouraging behaviors leading to water intoxication. This can lead to electrolyte imbalance, especially hyponatremia, causing symptoms such as abdominal pain, nausea, vomiting, myoclonic jerks, seizures, and delirium and may ultimately lead to coma and death [5]. PPD can be seen in 6-20% of psychiatric patients with 80% prevalence in schizophrenia [5]. The exact reasons for this association remain unclear, but it is thought to involve disturbances in brain mechanisms that control thirst and fluid balance, as well as possible influences from medications used to treat schizophrenia [6]. While schizophrenia itself carries a major burden for the patient and their families, it also poses a major issue in healthcare disparities for patients with psychiatric disorders. These disparities are complex and often affect access to appropriate medical care, treatment quality, and overall health outcomes. They can be influenced by socioeconomic status, ethnicity, and stigma related to mental illness [7]. In this case report, we present a patient with schizophrenia who presented with symptomatic hyponatremia secondary to PPD and, after many ED visits, was diagnosed with stage four uterine adenocarcinoma.

This article was previously presented as a meeting abstract and a poster presentation at the 2024 Rowan University Research Day on May 2, 2024, and as a poster presentation at the 2024 Atlantic Regional Osteopathic Conference on April 10, 2024.

## Case presentation

A 58-year-old Hispanic, White female with a significant past medical history of chronic schizophrenia complicated by multiple hospitalizations for hyponatremia secondary to PPD presented to the ED in February of 2016 with complaints of abdominal pain. The patient was well-controlled on Abilify Aristada long-acting injectable, Ativan, and Ambien for her significant psychiatric history and was predominantly Spanish-speaking. During this time, the patient endorsed command auditory hallucinations to ingest water and take over-the-counter (OTC) analgesics and laxatives to “rid of evil spirits” in her stomach. The patient consistently reported needing to remove the evil spirits she believed were in her stomach resulting in copious ingestion of water or OTC medications. Her complaints were ultimately attributed to a schizophrenic exacerbation and did not include robust medical workup, but rather discharge to follow-up outpatient psychiatry.

Over the next several years, the patient was readmitted seven times from 2020 to 2022 for two suspected aspirin overdoses, an Ambien overdose, and several visits to the ED. The initial overdose of aspirin resulted in hospitalization in December of 2020 where the patient presented with endorsement of abdominal pain and labs reporting significant hyponatremia, with a sodium of 103. Subsequent overdoses in April of 2021 (Ambien) and September of 2021 (Aspirin) also involved severe hyponatremia, with sodium levels of 100 and 102, respectively, with complaints of abdominal pain. An initial ultrasound of the abdomen was completed in September, revealing an incidental liver cyst and otherwise normal results. In December of 2021, she presented with nausea and vomiting, after drinking 5 to 6 liters of fluid over nine hours. Labs, including an iron panel, were collected, and the patient was found to have severe iron deficiency anemia.

**Figure 1:**
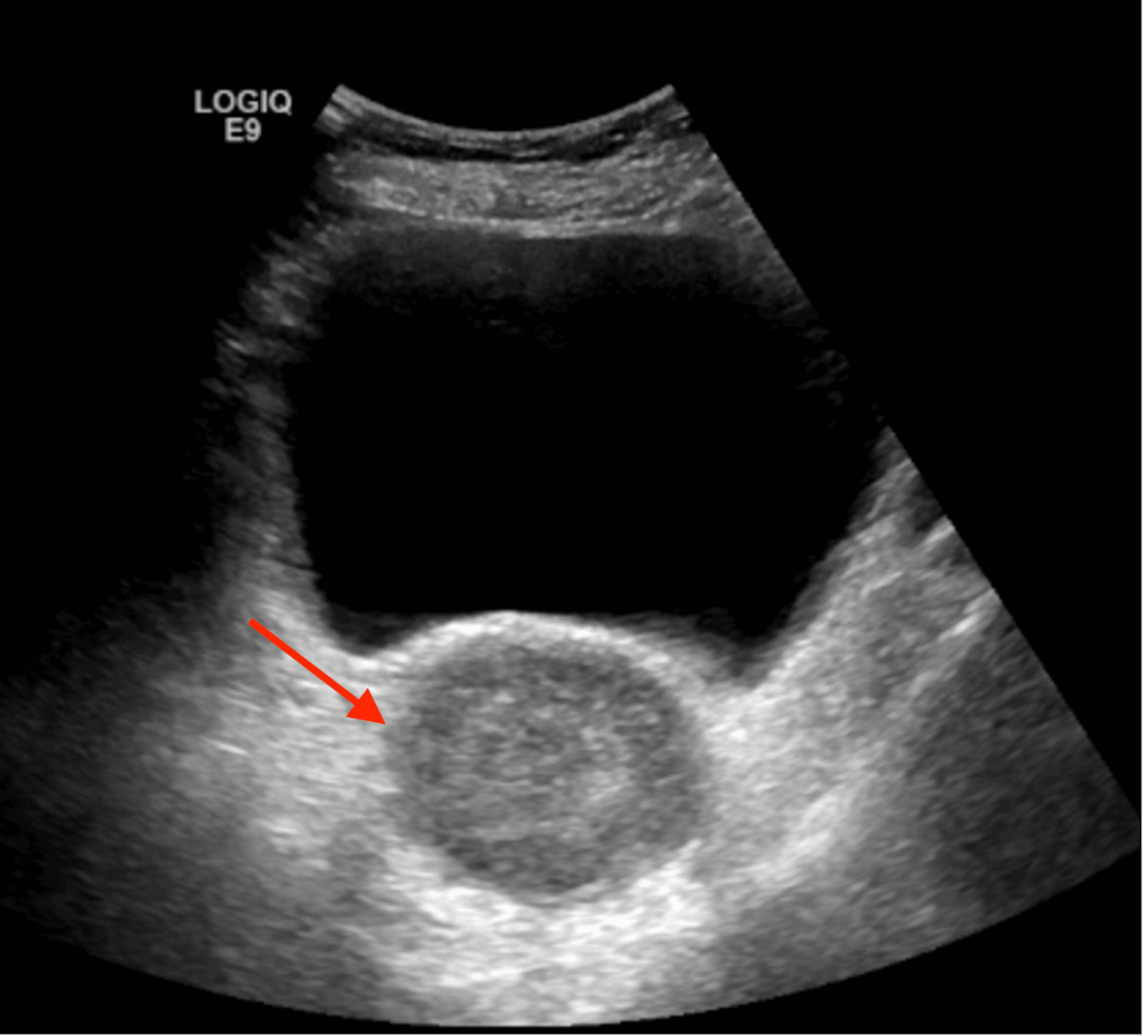
Pelvic ultrasound Infiltrative uterine mass

**Figure 2:**
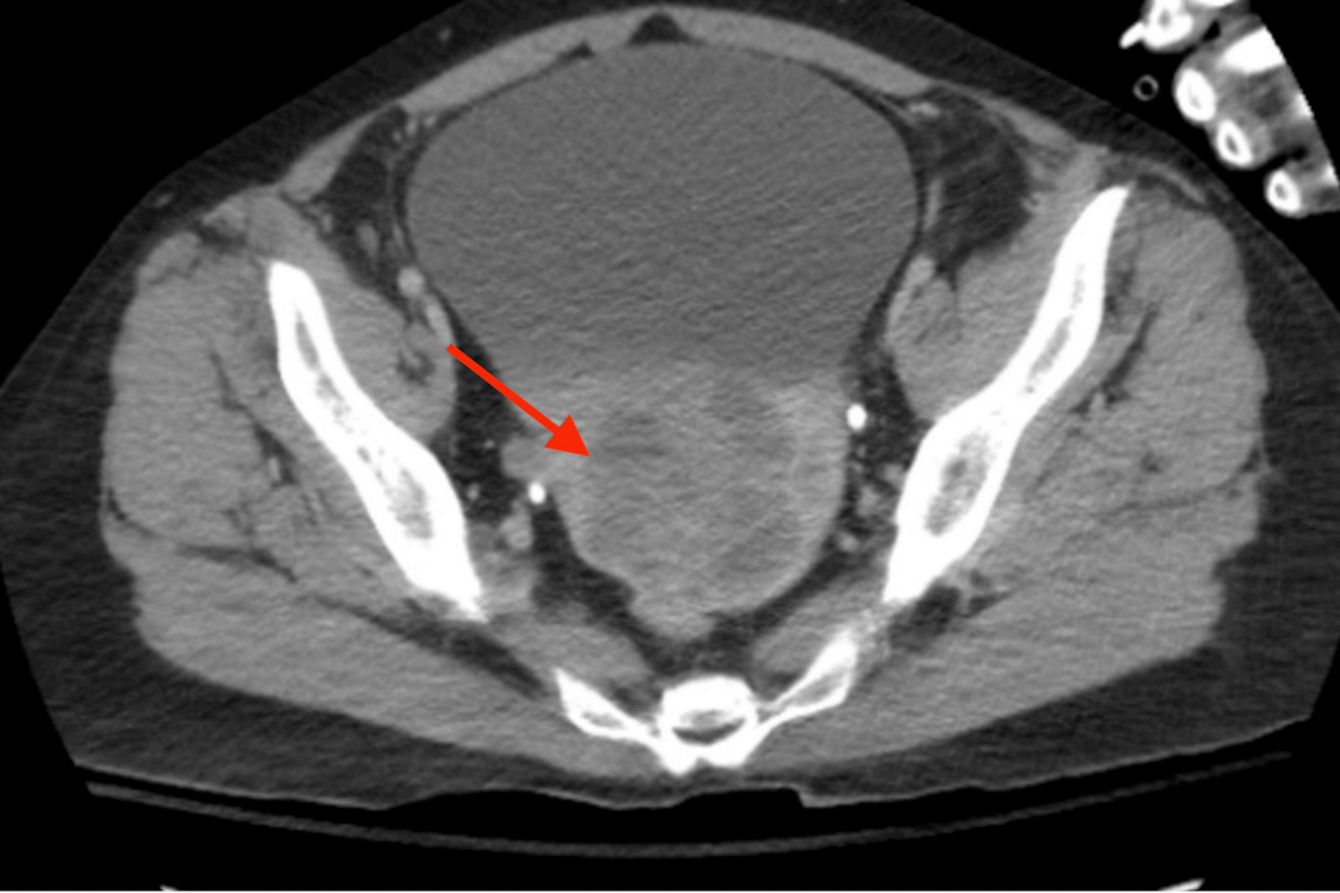
Axial CT abdomen and pelvis Heterogeneous irregular mass present within the uterus.

**Figure 3:**
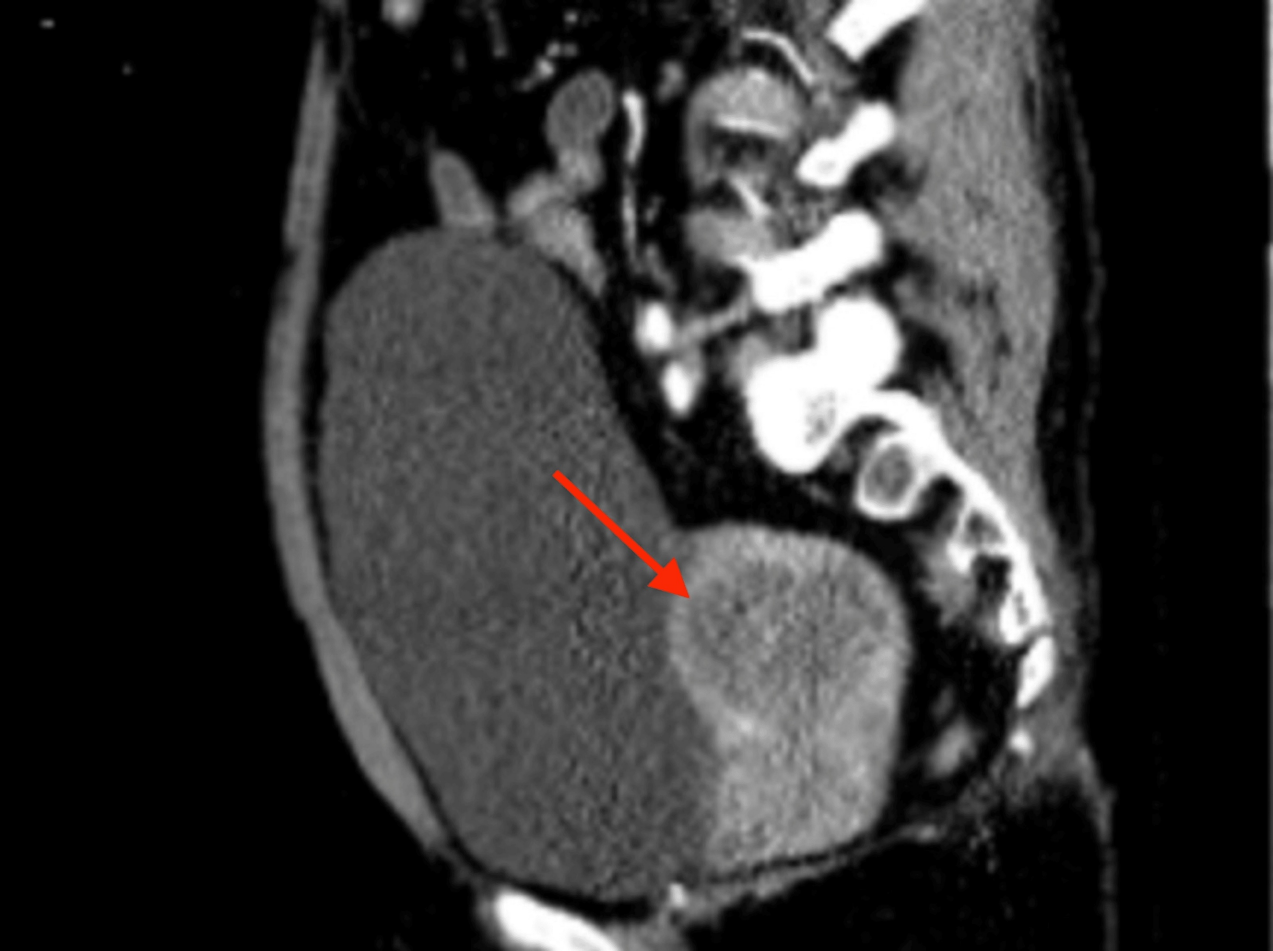
Sagittal CT abdomen and pelvis Hypoenhancing heterogeneous irregular mass within the uterus.

In February of 2022, the patient presented to the ED this time with diffuse abdominal pain accompanied by a headache, polydipsia, and polyuria for two days. She endorsed drinking six to seven glasses of water due to severe polydipsia and reported multiple episodes of vomiting, headache, dizziness, non-exertional shortness of breath, and fatigue concerning for worsening of her clinical course. She was found to be hypothermic, and labs reported microcytic anemia with a hemoglobin of 5.7. These results ultimately raised an underlying condition, for which tumor marker tests were collected providing abnormal results (CA125 85.9/50.1, CA19-9 >10, and CEA 0.3). Initial pelvic imaging with a pelvic ultrasound was completed, revealing an infiltrative uterine mass measuring up to 5.6 cm, and a gynecology oncology consult was recommended (Figure 1). A CT abdomen pelvis showed a hypoenhancing heterogeneous irregular mass within the uterus atypical per uterine fibroid and more suggestive of uterine cancer (Figures 2, 3). The patient was diagnosed with stage IV uterine adenocarcinoma via CT abdomen and pelvis and discharged home.

In July of 2022, the patient arrived at the ED complaining of lower abdominal pain, labs significant for sodium of 100, and vaginal bleeding for which gynecology was consulted. The patient was evaluated, and an MRI was recommended. However, ultimately, the patient refused the MRI and later would refuse a gynecology workup, uterine biopsy, and surgery given her advanced cancer.

## Discussion

This 58-year-old female was found to have advanced uterine adenocarcinoma masked by PPD secondary to schizophrenia. While she presented to the ED on multiple occasions with abdominal pain and OCP overdoses, the patient received insufficient workup of her abdominal pain, raising concern for insufficient patient care. Attributing her abdominal pain to hyponatremia secondary to PPD and ultimately schizophrenia led to premature closure bias, in which clinicians had come to a diagnosis or conclusion about a patient's condition quickly without further consideration of the cause [8]. This type of bias can lead to missed or delayed diagnoses, which was the circumstance in our case [8]. The patient's abdominal pain was immediately deemed a schizophrenia exacerbation on each presentation to the ED, in which further medical investigation was not completed, allowing for ultimately seven years of cancer development before proper diagnostic imaging was completed. 

Here, we demonstrate the consequences of healthcare disparities, especially in the psychiatric population. In this case, our patient not only faced these difficulties as a psychiatric patient but was also a part of an ethnic minority as a Hispanic, non-English-speaking patient with a language barrier. Differences in health quality and equity can manifest as poor access to healthcare services and adverse health outcomes [7]. These disparities often result from the interplay of social, economic, and environmental factors [7].

Patients with mental illness often face challenges in accessing primary care. While regular medical screening is often implemented and encouraged in the primary care setting, there has been a significant lag regarding implementing these measures in patients suffering from psychiatric illness [9]. It has been reported that approximately 50% of community mental health center patients have an established primary healthcare provider and 50% report access to proper referrals for appropriate healthcare providers and/or management for medical conditions [10]. This raises concerns that a large portion of the community suffering from mental health disorders lack access to primary care. Furthermore, when those patients do receive care in the primary setting, it is often of lesser quality [11], resulting in excessive emergency department utilization and lack of continuity of care. All of these lead to patient distrust of the healthcare system resulting in delayed treatment and worsening mortality. Our patient had no past history of cancer screenings, including colon, breast, and most importantly cervical, and never had a bimanual or pelvic exam completed. This raises the question of whether the patient's cancer might have been caught sooner with annual physical exams, cancer screening measures, and close medical follow-up. 

Furthermore, providers reported a general reluctance to treat individuals with severe mental illness including schizophrenia. Systemically, these patients are often seen as unfavorable due to insufficient insurance coverage and the extensive resources their care requires, such as longer visits and the need for case managers [9]. It has also been observed that medical providers often talk down to and make postulations about mental illness patients, provide less clear clarification about their care, and doubt their self-reported symptoms [10-11]. The patient discussed in this case report had endorsed abdominal pain and feelings of needing to expel evil from her body. While it is not known, it can be speculated that her complaints were her own way of attempting to share that something was wrong with her body. This highlights the need for more provider awareness of self-reported symptoms in psychiatric patients and a low index of suspicion in this particular population. This perceived negative attitude is often seen as a barrier to building rapport and trust, which we suspect contributed to our patient's choice not to proceed with treatment or further diagnostic tests after receiving her preliminary diagnosis.

## Conclusions

This patient highlights an example of a serious diagnosis that was delayed due to falsely attributing her symptoms to her psychiatric illness resulting in a lack of proper clinical investigation. Addressing healthcare disparities in patients with psychiatric disorders, especially those suffering from schizophrenia, necessitates a multidimensional approach that encompasses policy changes, cultural proficiency, collaboration and communication among interdisciplinary health professionals, and increased education and awareness. The patient discussed in this report was unfortunately an example of how healthcare disparities can lead to devastating consequences. By tackling these disparities, we can improve access to care, enhance treatment outcomes, and encourage overall health equity.

## References

[1] McCutcheon RA, Reis Marques T, Howes OD (2020). Schizophrenia-an overview. JAMA Psychiatry.

[2] Hany M, Rehman B, Rizvi A, Chapman J (2024). Schizophrenia. StatPearls [Internet].

[3] Hjorthøj C, Posselt CM, Nordentoft M (2021). Development over time of the population-attributable risk fraction for cannabis use disorder in schizophrenia in Denmark. JAMA Psychiatry.

[4] Hjorthøj C, Stürup AE, Mcgrath JJ, Nordentoft M (2017). Years of potential life lost and life expectancy in schizophrenia: a systematic review and meta-analysis. Lancet Psychiatry.

[5] Siddiqui J, Qureshi S, Abdulkhaliq A (2020). Psychogenic polydipsia leading to hyponatremia induced seizure in schizophrenia. J Mood Disord.

[6] Bhatia MS, Goyal A, Saha R, Doval N (2017). Psychogenic polydipsia - management challenges. Shanghai Arch Psychiatry.

[7] Khatri UG, Delgado MK, South E, Friedman A (2022). Racial disparities in the management of emergency department patients presenting with psychiatric disorders. Ann Epidemiol.

[8] Kumar B, Kanna B, Kumar S (2011). The pitfalls of premature closure: clinical decision-making in a case of aortic dissection. BMJ Case Rep.

[9] Kaufman EA, McDonell MG, Cristofalo MA, Ries RK (2012). Exploring barriers to primary care for patients with severe mental illness: frontline patient and provider accounts. Issues Ment Health Nurs.

[10] Druss BG, von Esenwein SA, Compton MT, Rask KJ, Zhao L, Parker RM (2010). A randomized trial of medical care management for community mental health settings: the Primary Care Access, Referral, and Evaluation (PCARE) study. Am J Psychiatry.

[11] Druss BG, Bradford WD, Rosenheck RA, Radford MJ, Krumholz HM (2001). Quality of medical care and excess mortality in older patients with mental disorders. Arch Gen Psychiatry.

